# IDO Targeting in Sarcoma: Biological and Clinical Implications

**DOI:** 10.3389/fimmu.2020.00274

**Published:** 2020-03-05

**Authors:** Imane Nafia, Maud Toulmonde, Doriane Bortolotto, Assia Chaibi, Dominique Bodet, Christophe Rey, Valerie Velasco, Claire B. Larmonier, Loïc Cerf, Julien Adam, François Le Loarer, Ariel Savina, Alban Bessede, Antoine Italiano

**Affiliations:** ^1^Explicyte Immuno-Oncology, Bordeaux, France; ^2^Department of Medical Oncology, Institut Bergonié, Bordeaux, France; ^3^Department of Pathology, Gustave Roussy, Villejuif, France; ^4^Institut Roche, Paris, France; ^5^Inserm U1218, Bordeaux, France

**Keywords:** sarcomas, immunotherapy, indoleamine, kynurenine, PDL1

## Abstract

Sarcomas are heterogeneous malignant mesenchymal neoplasms with limited sensitivity to immunotherapy. We recently demonstrated an increase in Kynurenine Pathway (KP) activity in the plasma of sarcoma patients treated with pembrolizumab. While the KP has already been described to favor immune escape through the degradation of L-Tryptophan and production of metabolites including L-Kynurenine, Indoleamine 2,3 dioxygenase (IDO1), a first rate-limiting enzyme of the KP, still represents an attractive therapeutic target, and its blockade had not yet been investigated in sarcomas. Using immunohistochemistry, IDO1 and CD8, expression profiles were addressed within 203 cases of human sarcomas. At a preclinical level, we investigated the modulation of the KP upon PDL1 blockade in a syngeneic model of sarcoma through mRNA quantification of key KP enzymes within the tumor. Furthermore, in order to evaluate the possible anti-tumor effect of IDO blockade in combination with PDL1 blockade, an innovative IDO inhibitor (GDC-0919) was used. Its effect was first assessed on Kynurenine to Tryptophan ratio at plasmatic level and also within the tumor. Following GDC-0919 treatment, alone or in combination with anti-PDL1 antibody, tumor growth, immune cell infiltration, and gene expression profiling were measured. IDO1 expression was observed in 39.1% of human sarcoma cases and was significantly higher in tumors with high CD8 infiltration. In the pre-clinical setting, blockade of PDL1 led to a strong anti-tumor effect and was associated with an intratumoral inflammatory cytokines signature driven by Ifng but also with a modulation of the KP enzymes including Ido1 and Ido2. IDO1 inhibition using GDC-0919 resulted in (i) a significant decrease of plasmatic Kynurenine to Tryptophan ratio and in (ii) a decrease of tumoral Kynurenine. However, GDC-0919 used alone or combined with anti-PDL1, did not show anti-tumoral activity and did not affect the tumor immune cell infiltrate. In order to elucidate the mechanism(s) underlying the lack of effect of GDC-0919, we analyzed the gene expression profile of intratumoral biopsies. Interestingly, we have found that GDC-0919 induced a downregulation of the expression of pvr and granzymes, and an upregulation of inhba and Dtx4 suggesting a potential role of the IDO pathway in the control of NK function.

## Introduction

Soft-tissue sarcomas (STS) represent a heterogeneous group of rare tumors including more than 70 different histological subtypes (1). The identification of new therapies for STS patients is of crucial importance. Indeed, 40 to 50% STS patients will develop metastatic disease. Once metastases are detected, the treatment is mainly based on palliative chemotherapy and median survival of patients in this setting is about 12 to 20 months (2).

Historically, sarcomas represent the first tumor model for which immunotherapy has been suggested as a relevant therapeutic strategy (3). The PD1-PDL1 interaction is a major pathway hijacked by tumors to suppress immune control. The normal function of PD1, expressed on the surface of activated T cells under healthy conditions, is to down-modulate unwanted or excessive immune responses, including autoimmune reactions. Recent studies have shown that PDL1 was expressed in 58% of cases of STS, osteosarcomas and GIST (4–7) and that this overexpression has been associated with poor prognosis (6, 7). Targeting the PD1/PDL1 interaction was associated with impressive anti-tumor activity in a pre-clinical model of osteosarcoma (8).

We and others have shown that PD1 targeting has only modest efficacy in patients with advanced STS (9–12). By analyzing tumor samples from patients treated with the PD1 antagonist Pembrolizumab, we observed a high level of IDO1 expression (12). IDO1 is a major regulator of the Tryptophan (Trp) catabolism pathway that has been linked to impairment of anti-tumor immunity and tumor growth in several models (13, 14). Kynurenine, which is notably produced by IDO1, is a key metabolite of this pathway that can promote selective expansion of regulatory T cells (Tregs) (15). Strikingly, we found a statistically significant increase in the kynurenine/tryptophan ratio, a robust pharmacodynamic marker of the IDO1/Kynurenine pathway (KP), between pre-treatment and on-treatment plasma samples of sarcoma patients treated with Pembrolizumab. Notably, this upregulation of the kynurenine/tryptophan ratio was also correlated with a higher density of IDO1 expression in pre-treated tumor samples (11).

IDO1/2 are major regulators of the KP, which is involved in immune homeostasis and tolerance—including fetomaternal tolerance—and avoiding acute and chronic hyperinflammatory reactions and autoimmunity. These enzymes induce biostatic tryptophan starvation, which limits lymphocyte expansion, and produce a series of catabolites, collectively known as kynurenines. L-kynurenine—the first, stable tryptophan catabolite in this pathway—induces T helper type-1 cell apoptosis and acts as an endogenous activator of the ligand-operated transcription factor aryl hydrocarbon receptor (AhR), thus promoting Treg cell generation and expansion. A novel oncotherapeutic approach, based on the inhibition of IDO1/2 by 1-methyl-tryptophan (1-MT) has demonstrated a synergistic efficacy when used in combination with conventional chemotherapy (16). Recent preclinical data have also evidenced that IDO1 deficient mice (Ido1–/–) were more sensitive to immune checkpoint inhibitors, including anti-CTLA4, anti-PD1, and anti-PDL1, thus highlighting the crucial role of IDO1 in resistance to those treatments. The same authors have shown that the prototypical IDO1 inhibitor, 1-MT, can synergize with the same immunotherapies (17).

Altogether, such data led us to hypothesize that the IDO1/KP pathway could preferentially contribute to the immune-suppressive phenotype of STS cells and be an important mechanism of the primary resistance to PD1 inhibition observed in sarcoma patients (8), and that targeting this pathway with an innovative compound would improve anti-PD1/PDL1 efficacy in this setting.

Currently, several investigational agents inhibiting the IDO-Kyn-Trp pathway such as IDO1, combination IDO1/TDO inhibitors, AhR inhibitors as well as a recombinant kynureninase are in clinical development or late preclinical testing (18). We investigated the pre-clinical activity of a specific IDO inhibitor—GDC-0919—and its capacity to synergize with PD1 inhibition in a preclinical model of STS.

## Results

### Histological Profiling Identifies Expression of IDO1 in Human STS

**Figure 1:**
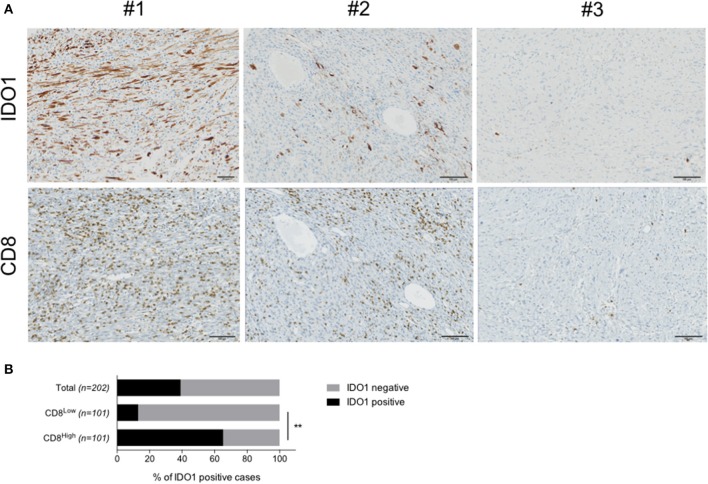
IDO1 is expressed in human sarcoma and its tumor microenvironment. **(A)** Immunohistochemical staining for IDO1 in a cases with positive tumor cells (#1), immune cells (#2) or no positivity (#3). Stainings for CD8 in sequential sections are shown. **(B)** Quantification of IDO1 positivity within the total cohort (*n* = 203), patients harboring either high (CD8^High^, *n* = 101) or low (CD8^Low^, *n* = 101) CD8 infiltration. Data are represented as percentage. **p < 0.01.

In order to investigate IDO1 expression in human STS, we performed an immunohistochemistry-based analysis for IDO1, PDL1, and CD8 markers of 203 cases of sarcomas with genomic complex (Figure 1A). Their characteristics are described in Table 1. The median density of CD8+ was 32.5 cells/mm^2^ (range 0–2663). Neither metastases-free survival nor overall survival was significantly different between patients with level of CD8 TL tumor density below or above the median (Supplementary Figure 1). IDO expression was observed in 79 cases (39.1%). The proportion of tumors with positive IDO1 staining was significantly higher in the group with higher levels of CD8+ TL tumor density than in the group with lower levels (65.3% vs. 12.9%, *p* < 0.001) (Figure 1B). Moreover, PDL1 expression was positive on tumor cells and on immune cells in 46 cases (22.7%), and almost exclusively in tumors with higher levels of CD8 TL tumor density (*data not shown*).

**Table 1:** Patient characteristics.

	**Nb of patients**	**%**
**Median age (min-max) 62 years (18-95)**		
**GENDER**		
Male	111	54.6
Female	92	45.4
**LOCATION**		
Limb	155	76.4
Trunk wall	21	10.4
Head and neck	2	1
Internal trunk	25	12.3
**Median tumor size (min-max) 97 mm (10-)**		
**HISTOLOGY**		
LPS	20	9.8
US/MFH	56	27.6
LMS	74	36.4
MYXOFIBROSARCOMA	30	14.8
Others	23	12.3
**GRADE**		
1	10	4.9
2	53	26.1
3	140	69.0
**DEPTH**		
Deep	145	71.5
Both superficial & deep	36	17.7
Superficial	22	10.8

### The KP Is Active in Human Sarcoma Cell Lines and Is Reversed by GDC-0919

**Figure 2:**
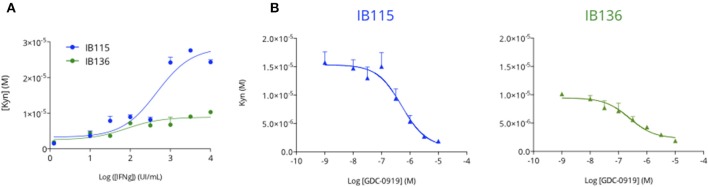
Primary human sarcoma cell lines display an inducible Tryptophan-degrading activity. **(A)** Two primary human sarcoma cell lines, IB115 and IB136, were treated with increasing concentrations of recombinant human Interferon gamma and cell culture supernatants were collected 48 h after treatment initiation for Kynurenine level assessment by mean of ELISA. **(B)** Both IB115 and IB136 were treated with a fixed concentration of Interferon gamma−10^3^U/mL and 10^4^U/mL respectively—and concomitantly treated with an increasing concentration of GDC-0919 (IDO inhibitor) for 48 h. Cell culture supernatants were processed for Kynurenine content measurement (ELISA). Means ± SEM are represented.

In order to evaluate the capacity of human sarcoma cells to produce Kynurenine as an immunosuppressive mechanism, we exposed two sarcoma cell lines—namely IB115 (dedifferentiated liposarcoma) and IB136 (leiomyosarcoma)—to increasing concentrations of human interferon gamma (hIFNg) for 48 h before cell supernatant collection for Kynurenine measurement by mean of an immuno-assay (ImmuSmol). We observed a hIFNg dose-dependent increase of Kynurenine, which was more pronounced for IB115 than for IB136 cell lines (Figure 2A). We then evaluated the capacity of the IDO1 inhibitor GDC-0919 to prevent from hIFNg-induced Kynurenine production. To this end, IB115 and IB136 cells were exposed to 1 × 10^3^ UI/mL and 1 × 10^4^ UI/mL hIFNg, respectively, in co-treatment with increasing concentrations of GDC-0919 (Figure 2B). As expected, treatment with GDC-0919 significantly inhibited the production of Kynurenine with IC_50_ values at 5.1 10^−7^M and 2.4 10^−7^M for IB115 and IB136 cell lines, respectively.

### MCA205 Sarcoma Preclinical Model Harbors KP Activity

**Figure 3:**
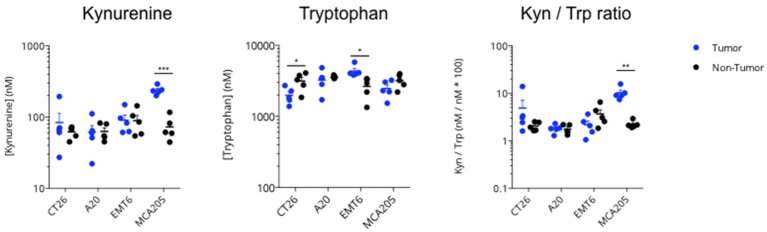
Intratumoral activity of the KP in several syngeneic mouse models. Four different syngeneic mouse models—CT26 colon cancer, A20 lymphoma, EMT6 breast cancer, and MCA205 sarcoma—were processed at an average tumor volume of 200 mm^3^, for non-tumor (contralateral) and intratumoral microdialysis. Collected microdialysates were then processed for simultaneous measurement of Kynurenine and Tryptophan content by LC/MS; Kynurenine to Tryptophan ratio was then calculated. For statistical analysis, student *t*-test was used. *p < 0.05, **p < 0.01, ***p < 0.001. Means ± SEM are represented.

In order to characterize the respective levels of activity of the IDO-Kyn-Trp pathway in different syngeneic mouse tumor models, we quantified Kynurenine and Tryptophan levels in the extracellular space of the tumor using intratumoral microdialysis in each of the following models: CT26 colon cancer, A20 lymphoma, EMT6 breast cancer, and MC205 sarcoma models. Microdialysis in a “non-tumor bearing” area was performed on the opposite flank and served as a control. As depicted in Figure 3, MCA205 mouse model displayed the highest level of Kynurenine to Tryptophan ratio with a significant difference between tumor and non-tumor regions thus suggesting the expression and activity of Tryptophan-degrading enzymes.

### Modulation of the KP Upon Anti-PDL1 Treatment in the MCA205 Mouse Model

**Figure 4:**
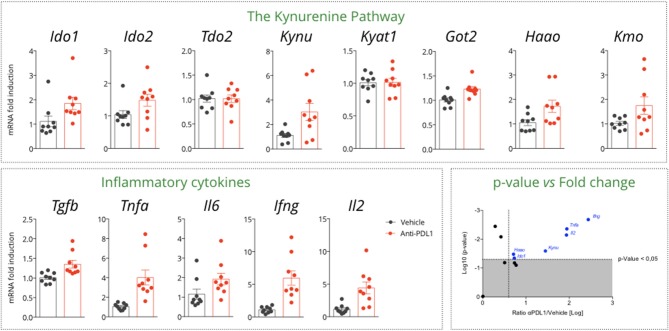
Gene expression assessment in tumor samples collected from a MCA205 tumor-bearing model treated or not with anti-PDL1. Tumor samples were retrieved 13 days post tumor inoculation, and subjected to RT-qPCR for expression analysis of genes encoding for the different enzymes of the KP and key inflammatory cytokines. On the right bottom, display of the *p*-values vs. the log transformed ratio of tumor mRNA level between PDL1 and Vehicle. Means ± SEM are represented. *Ido1*, Indoleamine 2,3 dioxygenase 1; *Ido2*, Indoleamine 2,3 dioxygenase 2; *Tdo2*, Tryptophan dioxygenase; *Kynu*, Kynureninase; *Kyat1*; Kynurenine Aminotransferase 1; *Got2*, Kynurenine Aminotransferase 4; *Haao*, Hydroxyananthranilate oxygenase; *Kmo*, Kynurenine Monooxygenase.

We then investigated the gene expression profiles of KP and immune-related genes in MCA205 tumor biopsies collected on vehicle and anti-PDL1 treated mice 13 days post tumor inoculation. Interestingly, we observed an increase in Tryptophan-degrading enzymes upon PDL1 blockade—*Ido1* and *Ido2—*while no change was observed for *Tdo2* (Figure 4). Correlation studies between genes encoding for KP enzymes and immune markers pointed that *Ido1* level was correlated to Il6 (*R*^2^ = 0.69) and to a lesser extent to Ifng (*R*^2^ = 0.36) (Supplementary Figure 2). Besides *Ido1* and *Ido2*, we also observed an induction of genes encoding downstream enzymes in the IDO-KYN-TRP pathway such as Kynureninase (*Kynu*) or kynurenine monooxygenase (*Kmo*) which converts Kynurenine to Anthranilic acid or to 3-hydroxyKynurenine, respectively. Hydroxyanthranilate oxygenase (*Haao*) favoring the degradation of the immunosuppressive metabolite Hydroxyanthranilic acid was also significantly upregulated (Figure 4) thus highlighting a global effect of PDL1 blockade on the pathway. As expected, anti-PDL1 favored the induction of an immune response within the tumor as highlighted by *Tnfa, Il6, Ifng, Il2*, and *Tgfb* induction—Ifng expression levels being significantly correlated with survival (*Data not shown*). Tumor-draining lymph nodes (TDLNs) are a critical compartment for the control of the anti-tumor immune response. We thus investigated the expression level of the same genes in this compartment, which only revealed a non-significant induction of Ido1. The other enzymes of the pathway were not affected (Supplementary Figure 3).

### Treatment With IDO Inhibitor Limits Kynurenine Production

**Figure 5:**
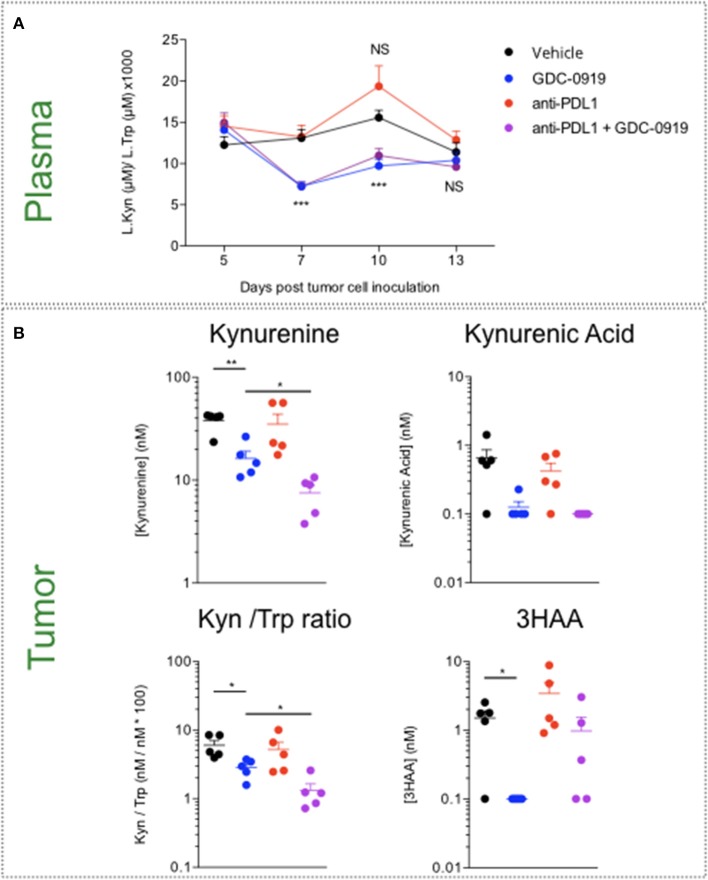
Assessment of Tryptophan catabolism at both peripheral and tumor levels upon pharmacological inhibition of IDO1 and PDL1 blockade. MCA205 tumor-bearing mice were treated with either GDC-0919, anti-PDL1, or combination of both. **(A)** Mice were bled at different time-points following tumor inoculation, and Kynurenine to Tryptophan ratio was analyzed by mean of ELISA. **(B)** Thirteen days post tumor inoculation, animals from all the experimental groups were processed for intratumoral microdialysis and several metabolites of the KP—Kynurenine, Tryptophan, Kynurenine to Tryptophan ratio, Kynurenic Acid, and 3-OH Anthranilic acid—were quantified in the collected dialysates by LC/MS. For statistical analysis, student *t*-test was used. *p < 0.05, **p < 0.01, ***p < 0.001, NS, not significant. Means ± SEM are represented. In A, experimental groups are compared to Vehicle-treated animals.

Before investigating the benefit of IDO inhibition in the preclinical sarcoma model, we evaluated the impact of GDC-0919—a novel IDO1 inhibitor—on Kynurenine and Tryptophan levels both at peripheral and tumoral levels. Two doses of GDC-0919−100 and 200mg/kg, po, bid—were explored in a LPS model known to promote Kynurenine production. As expected, Kyn/Trp ratio increase was induced upon LPS administration and was reversed in a same extent with 100 and 200mg/kg of GDC-0919 (Supplementary Figure 4). Treatment of MCA205 tumor bearing mice with GDC-0919 either as a single agent (100mg/kg, po, bid) or in combination with an anti-PDL1 antibody yielded a significant decrease in plasmatic Kynurenine to Tryptophan ratio. However, this event was only transient as no significant difference was observed at the latest time-points (ie., Day 13 post tumor inoculation) (Figure 5). By using intratumoral microdialysis, we evaluated the effect of GDC-0919 on the tumoral extracellular contents of kynurenine pathway metabolites 13 days after tumor cell inoculation. As expected, GDC-0919 induced a substantial decrease in Kynurenine, Kynurenine to Tryptophan ratio, and Kynurenic acid. Interestingly, combining an anti-PDL1 antibody to GDC-0919 favored the decrease of upstream metabolites (Kynurenine and Kynurenic acid) while partially preventing the decrease in 3HAA, a downstream metabolite of the pathway (Figure 5).

### IDO Inhibition Does Not Optimize Anti-PDL1 Mediated Anti-tumor Response

**Figure 6:**
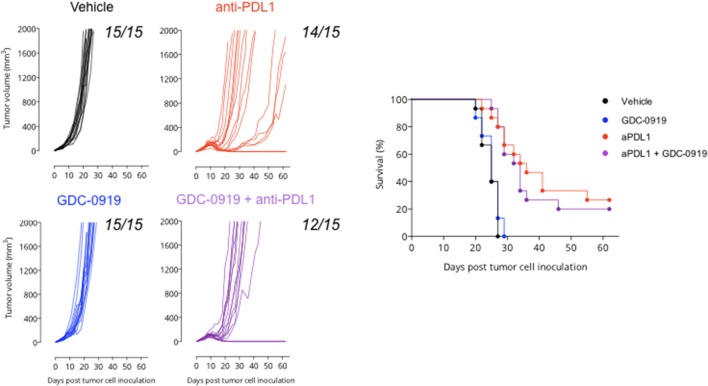
Anti-tumor assessment of IDO1 blockade in the preclinical sarcoma model. MCA205 sarcoma cell line was injected subcutaneously and animals were then treated or not (Vehicle) with either (i) anti-PDL1 starting from day 6 post tumor inoculation and then repeated every 3 days for a total of 4 injections or (ii) GDC-0919 starting from day 6 post tumor inoculation and then treated orally twice daily or combination of both (as described in Material and Methods). Tumor growth kinetics (left panel), and survival curves (right panel) are depicted. Each graph represents 1 experiment with 15 animals per group.

**Figure 7:**
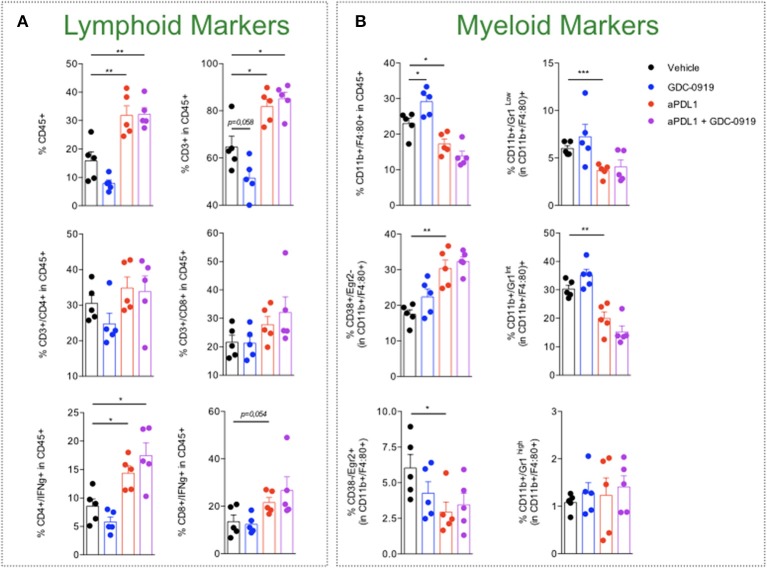
Characterization of tumor-infiltrating leukocytes upon KP blockade. MCA205 sarcoma cell line was subcutaneously inoculated and animals were then treated or not (Vehicle) with either (i) anti-PDL1 or (ii) GDC-0919 or combination of both (as described in Material and Methods). Thirteen days after tumor inoculation, tumors were retrieved and processed for cell suspension preparation and then analyzed by flow cytometry for **(A)** lymphoid or **(B)** myeloid markers. Student t-test was used. *p < 0.05, **p < 0.01, ***p < 0.001, n.s.: not significant. Means ± SEM are represented.

As depicted by tumor growth assessment, anti-PDL1 administration triggered a significant anti-tumor response resulting in 1 out of 15 tumor rejection. IDO1 blockade using GDC-0919 did not confer significant benefit either alone or in combination with anti-PDL1—even if a trend of optimization was observed with a tumor rejection observed on 3 out of 15 mice tested (Figure 6). In order to investigate the *in vivo* mechanism of action of the different treatment regimens, tumor infiltrating leukocytes (TILs) were assessed through extensive flow cytometry analysis (Figure 7). As expected, PDL1 blockade favored tumor infiltration by CD45+ leukocytes, mainly composed of a higher fraction of CD4+ and CD8+ T cells also harboring a more pronounced activated state as depicted by intracellular IFNgamma staining. Combination of anti-PDL1 with GDC-0919 did not change the observed features. Administration of GDC-0919 alone limited the tumor proportions of CD3+ lymphocytes and CD4+ T cells, which were also shown to be less activated based on the IFNgamma staining. As to the myeloid cell subsets, anti-PDL1 limited macrophages infiltration (CD11b+/F4/80+ cells) and concomitantly **i)** favored a M1 state – defined as CD38+/Egr2- and **ii)** limited the M2 immunosuppressive cell subset (CD38-/Egr2+). Unlike anti-PDL1, GDC-0919 used as a single agent favored macrophages infiltration of the tumor. Combining GDC-0919 to the anti-PDL1 antibody did not result in significant changes in comparison to anti-PDL1 used as a single agent. Analysis of Myeloid Derived Suppressor Cells (MDSCs) by mean of CD11b and Gr1 staining revealed that anti-PDL1 limited their infiltration within the tumor – especially CD11b+/Gr1^Low^ and CD11b+/Gr1^Int^ populations. No changes in CD11b+/Gr1^High^ were observed. GDC-0919 had no impact on these cell populations, neither alone nor in combination with anti-PDL1.

### Gene Expression Modulation Upon GDC-0919 Treatment

**Figure 8:**
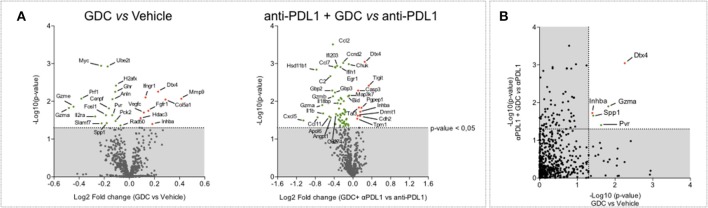
Highlight of possible resistance mechanisms to IDO1 blockade through tumor gene expression profiling. **(A)** Volcano plot of NanoString gene expression analysis in tumor samples comparing GDC-0919 vs. Vehicle groups (left panel) and GDC-0919 + anti-PDL1 vs. anti-PDL1 (right panel). **(B)** Plot representation of *p*-value comparing GDC-0919 vs. Vehicle groups (X axis) and GDC-0919 + anti-PDL1 vs. anti-PDL1 (Y axis). Green dots indicate downregulations; Red dots indicate upregulations. Dashed line depicts a 0,05 significativity.

In order to characterize the molecular response induced by IDO1 blockade, intratumoral biopsies—collected 13 days post tumor inoculation—were subjected to a gene expression analysis using Nanostring technology and the PanIO360 panel. As expected, PDL1 blockade was shown to strongly modulate different genes belonging to several pathways including interferon signaling (Supplementary Figure 5). GDC-0919 applied alone favored the modulation of several genes including the downregulation of Gzma and Gzme (encoding for Granzyme A and E, respectively) but also Prf1 (encoding for Perforin) (Figure 8A). When combined with anti-PDL1, GDC-0919 triggered several gene expression changes, including the upregulation of the inhibitory molecule Tigit that could participate in the lack of synergistic effect of GDC-0919 and anti-PDL1. As to identify genes specifically modulated upon GDC-0919—either alone or combined with anti-PDL1—we assessed genes for which a significant difference has been observed in GDC-0919 vs. Vehicle and anti-PDL1 vs. anti-PDL1 + GDC-0919. Interestingly, only a few genes were found significantly modulated in both situations (Figure 8B) including a downregulation of Granzyme a, Pvr (encoding for the poliovirus receptor, CD155), and Spp1 (encoding for the Osteopontin) and an upregulation of inhba, encoding for the inhibin—a member of the Tgfbeta signaling, and the E3 Ubiquitin ligase Dtx4.

## Discussion

Despite the revolution of cancer immunotherapy revealed by the use of immune checkpoint blockers, it still remains a challenge to optimize clinical response through combination therapies targeting resistance mechanisms. Considering preclinical and clinical evidence, blockade of IDO-driven tryptophan catabolism represents an exciting therapeutic avenue to potentiate cancer immunotherapy for which several candidates have been developed and tested in different clinical settings (19). We have recently demonstrated a modulation of the KP at the peripheral level in the PEMBROSARC trial assessing the efficacy of Pembrolizumab in STS patients, thus highlighting this pathway as a possible and relevant resistance mechanism to PD1 inhibition in STS (12).

We investigated here a large cohort of human STS for several immune markers (CD8, IDO1, PDL1). High CD8+ TL infiltration has been associated with favorable prognosis in different malignancies (20). In our series, neither metastases-free survival nor overall survival was significantly different between patients with level of CD8 TL tumor density below or above the median, or IDO1 positive or negative tumors. However, the proportion of tumors with positive IDO1 staining was significantly higher in the group with higher levels of CD8+ TL tumor density than in the group with lower levels. Moreover, PDL1 expression was positive in tumor cells in seven (12%) and in immune cells in 26 (44%) of the 59 samples analyzed, and almost exclusively in tumors with higher levels of CD8 TL tumor density. These findings are in line with our previous work showing IDO1 expression as a frequent hallmark of STS (12). Altogether, these data suggest a peculiar role of the IDO pathway in sarcoma progression and thus therapeutic potential of IDO inhibitors in a subset of sarcomas.

Here, by using a preclinical model of fibrosarcoma, we evaluated the benefit on *in vitro* and *in vivo* anti-tumor response of IDO1 blockade through a pharmacological approach using GDC-0919 alone or in combination with an immune checkpoint blocker. This IDO1 inhibitor demonstrated (i) a good pharmacological activity *in vitro* on human cell lines and (ii) the expected *in vivo* pharmacodynamic profile with peripheral and intratumoral decrease of Kynurenine. These results are consistent with those reported from the phase I study investigating GDC-0919 as a single agent and in combination with Atezolizumab in patients with advanced solid tumors (21). Indeed, GDC-0919 significantly reduced plasma Kynurenine in comparison to pre-dose levels. However, we observed that GDC-0919 alone or combined with anti-PDL1, only yielded to a transient decrease in plasmatic Kynurenine level thus suggesting a delayed compensatory mechanism restoring in turn Kynurenine production. As to investigate whether this feature is also observable within the tumor compartment, a longitudinal intratumoral microdialysis could be performed. Also, in future pre-clinical and/or clinical studies investigating the impact of IDO1 blockade, expression assessment of the other tryptophan degrading enzymes (IDO2, TDO2) could be evaluated. Nevertheless, whether alone or combined with anti-PDL1, GDC-0919 did not show anti-tumoral activity and did not significantly affect the tumor immune cell infiltrate, thereby corroborating the data obtained in patients. Indeed, the overall response rates observed in a cohort of 75 patients treated with GDC-0919 and Atezolizumab were not significantly different from those expected with Atezolizumab alone (21). Moreover, serial tumor biopsies did not show any significant intratumoral increase in CD8 or tumor infiltrating leukocytes (21).

In order to elucidate the mechanism(s) underlying the lack of effect of GDC-0919, intratumoral biopsies were performed and subjected to gene expression profiling and analysis under GDC-0919 alone and in combination with anti-PDL1. Interestingly, we have shown that the IDO1 inhibitor either as single agent or combined with anti-PDL1 significantly induced a downregulation of the expression of pvr and granzymes, and an upregulation of inhba and Dtx4. Inhba is a ligand in the transforming growth factor β (TGF-β) superfamily, which has been shown to regulate Ido1 via the Smad signaling pathway (22). A recent study has demonstrated that inhba plays a crucial role in inhibiting NK cell proliferation and production of granzyme B thereby leading to impairment of tumor susceptibility to NK cell-mediated killing (23). Finally, the Dtx4 E3-Ubiquitin ligase—known to promote TBK1 degradation and Type 1 interferon response (24)—was found to be upregulated. Evidences from the literature support the implication of NK cells in the anti-tumor activity of PD1/PDL1 blockers (25). Altogether, our data suggest a potential role for inhibitors of the IDO pathway in the down-regulation of NK function, which could explain why IDO inhibition did not result in a synergistic effect when combined with PD1/PDL1 blockers. These new evidences thus warrants further investigation to delineate the mechanism of action underlying the impact of GDC-0919 on NK cell activity toward tumor cells and calls the interest to analyze NK cell features/biology in cancer patients undergoing treatment regimen combining immune checkpoint blockers (ICB) with IDO inhibitors—all of these to give insights in order to design novel therapeutic strategies improving ICB efficacy.

## Materials and Methods

### Human Tumor Samples

All Formalin Fixed Paraffin Embedded (FFPE) tumors used in this work were collected at the Institut Bergonié (Bordeaux, France). For each case, tumor histology was confirmed by an experienced pathologist before staining processing.

### Study Approval

The study was approved by the Institutional Review Board of the Institut Bergonié (Bordeaux, France), and the methods were carried out in accordance with the approved guidelines and with written informed consent from all patients. All animal experiments were performed with the approval of the Institutional Animal Use and Care Committee.

### Immunohistochemistry

FFPE specimens were processed for immunohistochemistry with respective antibodies specific for IDO1 (clone UMAB126, Origen), CD8 (clone SP16, Spring Bioscience), and anti-PDL1 (clone SP263, Ventana) according to conventional protocols using a Ventana Benchmark Ultra automated platform Briefly, tissue sections were deparaffinized, and processed for epitope retrieval in CC1 buffer. After primary antibody incubation, amplification and detection steps used either an UltraView or an OptiView kit, DAB was used as a chromogen and counterstaining was performed with haematoxylin. Brightfield images were acquired using a VS120 virtual slides platform (Olympus). CD8+ cells density was evaluated using a dedicated image analysis algorithm as previously described (26). IDO1 staining was scored semi-quantitatively in tumor cells taking into account both the percentage of positive cells and their intensity from 0 to 3). Expression of PD-L1 in tumor cells (percentage of tumor cells stained) and immune cells (percentage of tumor area occupied by PD-L1 positive immune cells) was evaluated by a trained pathologist (JA). Positivity was defined as ≥1% either in tumor cells or immune cells.

### Cell Culture

The human cell lines used in this study were derived from human surgical STS specimens collected at the Institut Bergonié (Bordeaux, France) after obtaining patient consent [16]. All cell lines were cultured in RPMI 1640 medium containing GlutaMAX™ supplement (Life Technologies), 10% (*v*/*v*) fetal bovine serum (FBS), 1% penicillin/streptomycin and 0.2% Normocin (InvivoGen) at 37°C with 5% CO2. Murine cell lines—A20, 4T1, CT26, and MCA205—used for syngeneic mouse models were cultured in their appropriate media according to ATCC specifications. Cell lines were checked for their mycoplasma-free status before use.

### Animal Models

All mice were housed in A2 animal facility. All animal studies were carried out under protocols approved by the Institutional Animal Care and Use Committee at University of Bordeaux. Colon CT26, Lymphoma A20, Breast EMT6, and Sarcoma MCA205 cancer cell lines were cultured *in vitro* according to ATCC specifications, and were checked for their mycoplasma-free status before use. A cell suspension was prepared according to the viable cell count and was inoculated in either Balb/c (CT26, A20, and EMT6) or C57BL/6J (MCA205) mice purchased from Charles River (L'Abresle, France). Anti-PD-L1 monoclonal antibody (a-PDL1, clone 10F9-G2; purchased from BioXCell) was applied intraperitoneally at 5mg/kg and treatment was repeated 4 times, on days 6, 9, 12, and 15. GDC-0919 was freshly prepared every day in 1% Carboxymethylcellulose prepared in order to administer 100mg/kg twice daily. Treatment was applied from day 6 to day 26 post tumor inoculation (21-days treatment duration). Starting from day 6 post MCA205 tumor cell inoculation, all experimental animal groups were monitored 3 times a week for tumor volume measured by physical examination and according to the formula “V = Length ^*^ Width2/2.” In order to perform kynurenine and Tryptophan level assessment in plasma, animals were bled on days 5, 7, 10, and 13 post tumor inoculation, ~2 h after the morning treatments. Blood was collected by retroorbital venipuncture using EDTA tubes (Microvette, Sarstedt), and samples were then processed for Kynurenine and Tryptophan dosage by ELISA (ImmuSmol, #ISE-2227).

### Intratumoral Microdialysis

Thirteen days post tumor cell inoculation, mice from satellite group were anesthetized by isoflurane inhalation (Compact Anesthesia module, MINERVE) and connected to the microdialysis platform (CMA microdialysis, Sweden). A microdialysis probe (CMA) was inserted within the tumor and, unless indicated in the controlateral non-tumor region, and flushed continuously with a microperfusion buffer prior to the collection of the dialysates. After this stabilization period, dialysate collection was performed over 60 min. Tubes were stored at −80°C until metabolite dosage by LC/MS including L-Tryptophan, L-Kynurenine, Kynurenic acid, 3-Hydroxyanthranilic acid.

### KP Metabolites Quantification by Liquid Chromatography Coupled To Mass Spectrometry (LC/MS)

Five microliter of microdialysate samples were diluted in 45μL of acetonitrile. Diluted samples were vortexed and analyzed by LC/MS system consisting of a Waters ACQUITY UPLC® equipped with an UPLC CSH column and coupled to a Waters XEVO™ TQ-XS Mass Spectrometer (Waters Corporation, Milford, MA, USA) operating in the positive ion electrospray multiple reaction monitoring mode. Quantification was performed using standard calibration curves and internal labeled standards.

### Digital Multiplexed Gene Expression Analysis

Tumor biopsy samples were collected from syngeneic sarcoma MCA205 tumor-bearing mice 13 days after tumor cell inoculation, further homogenized in Trizol (Fermentas, Fisher Scientific SAS; Illkirch, France) and RNA was isolated using a standard chloroform/isopropanol protocol (27). After RNA extraction, DNAse digestion step and RNA control quality, extracted RNAs were dosed using microvolume spectrophotometry by Nanodrop 8000 platform (ThermoFisher, Waltham, MA, USA). RNA samples were subsequently diluted in RNase-free water to a concentration of 20 ng/μL. A Reporter CodeSet MasterMix was created by adding 70 μL hybridization buffer to the PanCancer IO 360™ Gene Expression Panel [Probeset XT_Mm_IO360 (NanoString Technologies, Seattle, WA, USA)]. Hybridization reactions of 100 ng RNA were incubated for 24 h at 65°C and ramped down to 4°C followed by digital barcode counting using NanoString nCounter Digital Analyzer (NanoString Technologies, Seattle, WA, USA). Background thresholding was fixed to count value of 20. The geometric mean of positive controls was used to compute positive control normalization parameters. Samples with normalization factors outside 0.3–3.0 were excluded. The geometric mean of housekeeping genes was used to compute the reference normalization factor. Samples with reference factors outside the 0.10–10.0 range were also excluded. Analysis of gene expression was done using the Advanced Analysis module on the nSolver software (NanoString Technologies, Seattle, WA, USA).

### RTqPCR Gene Expression Analysis

Cryopreserved tumor biopsies were subjected to tissue preparation procedure by tissue lysis and stainless steel beads prior to RNA extraction according to a Trizol protocol. After RNA extraction, samples followed a DNAse digestion step, RNA control quality on Bioanalyzer 2100 (Agilent, Santa Clara, CA, USA) and quantification on Nanodrop 1000 spectrophotometer (ThermoFisher, Waltham, MA, USA). cDNAs were prepared using PowerAid Premium reverse Transcriptase enzyme (ThermoFisher, Waltham, MA, USA). qPCR procedure was performed using LightCycler 480 (Roche Diagnostics, Mannheim, Germany) in Sybr green (Life Technologies Corp., Carlsbad, CA, USA), primers were designed and validated. Reference genes were selected with GeNorm algorithm.

### Flow Cytometry for Immune Cell Infiltrate Analysis

At day 13 post tumor cell inoculation, satellite animals from each experimental groups were sacrificed, and tumors were collected. Tumors were processed for organ dissociation using the GentleMACS system (Miltenyi Biotec). Tumor homogenates were then processed for immunostaining and profiling analysis by flow cytometry for the (i) lymphoïd panel—Viability marker/CD45/CD3/CD4/CD8/IFNg, and (ii) myeloid panel—Viability marker/CD45/CD11b/F4:80/CD38/Egr2/Gr1. For the lymphoid panel analysis and in particular for IFNgamma, cells were first stimulated with a cocktail of PMA, Ionomycin and Brefeldin A.

### Statistical Analysis

Two tailed Student's *t*-test was utilized to derive statistical significance. The minimal level of significance was *p* < 0.05 (Graph PadPRISM version 6, La Jolla, California, USA).

## Data Availability Statement

The raw data supporting the conclusions of this article will be made available by the authors, without undue reservation, to any qualified researcher.

## Ethics Statement

The studies involving human participants were reviewed and approved by the Institutional Review Board of the Institut Bergonié. The patients/participants provided their written informed consent to participate in this study. The animal study was reviewed and approved by the Ethic Committee on animal testing from Bordeaux University.

## Author Contributions

IN, AI, and AS, designed and supervised the study as a whole. MT, JA, and FL designed and supervised the histological part. CR and VV performed the immunohistochemistry. AC performed the TILs analysis. DBor performed the majority of *in-vivo* experiments. DBod performed the Kynurenine and Tryptophan dosage by ELISA. LC and CL performed analyses of gene expression data. AB and AI wrote the manuscript.

### Conflict of Interest

AB, IN, DBor, AC, DBod, CR, and LC are employed by the company ImmuSmol/Explicyte Immuno-Oncology. AS was employed by Institut Roche SAS. JA is member of the advisory boards of AstraZeneca, Bayer, BMS, MSD, Pfizer, Pierre Fabre, Roche, Sanofi. AI receives research grants from AstraZeneca, Bayer, BMS, MSD Pharmamar, Roche and is an advisory board member of Bayer, Daiichi, Epizyme, Lilly, MSD, Roche, Springworks. The remaining authors declare that this study received funding from Institut Roche (Boulogne-Billancourt, France) and Explicyte Immuno-Oncology (Bordeaux, France). The funders had the following involvement with the study: manuscript writing.
