# Supplementary figures and images for: IDO Targeting in Sarcoma: Biological and Clinical Implications
Source: Front Immunol. 2020 Mar 5;11:274. doi: 10.3389/fimmu.2020.00274 (PMC7066301; doi:10.3389/fimmu.2020.00274)

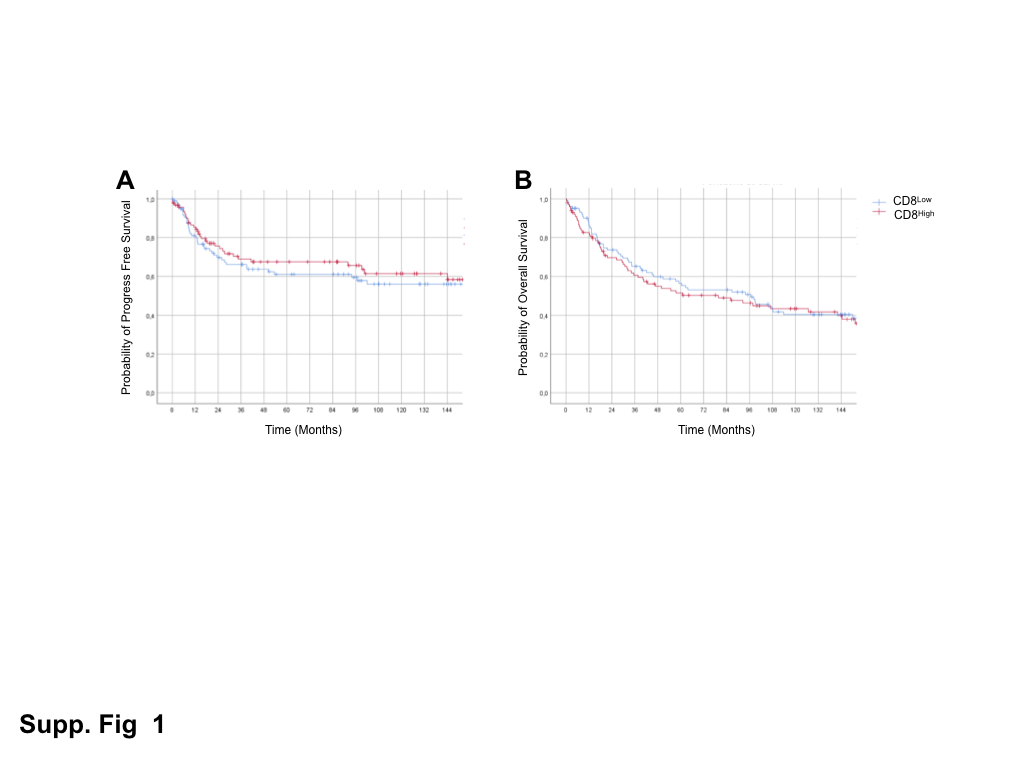

Supplement: Supplementary file 1 [file Image_1.tiff]

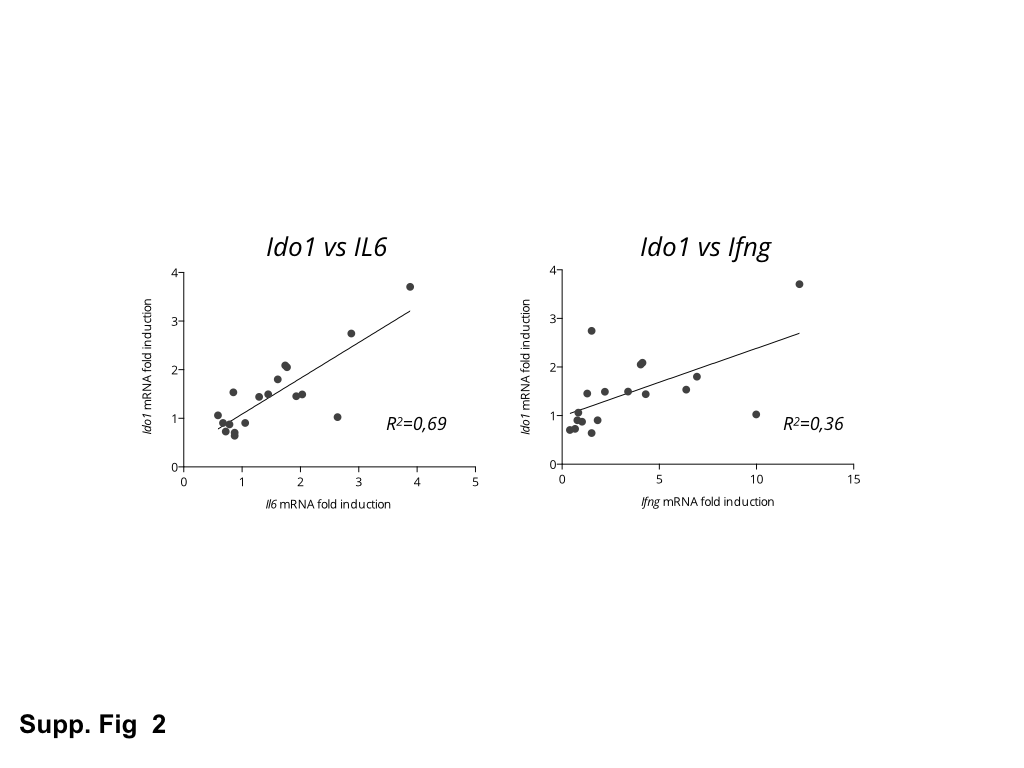

Supplement: Supplementary file 2 [file Image_2.tiff]

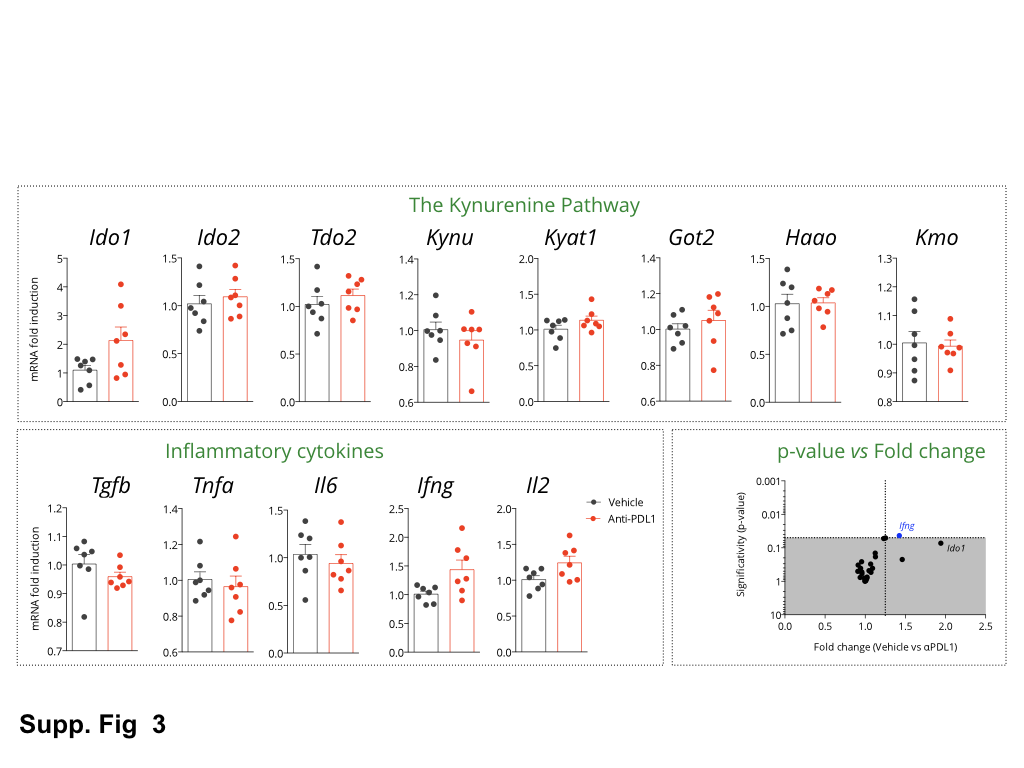

Supplement: Supplementary file 3 [file Image_3.tiff]

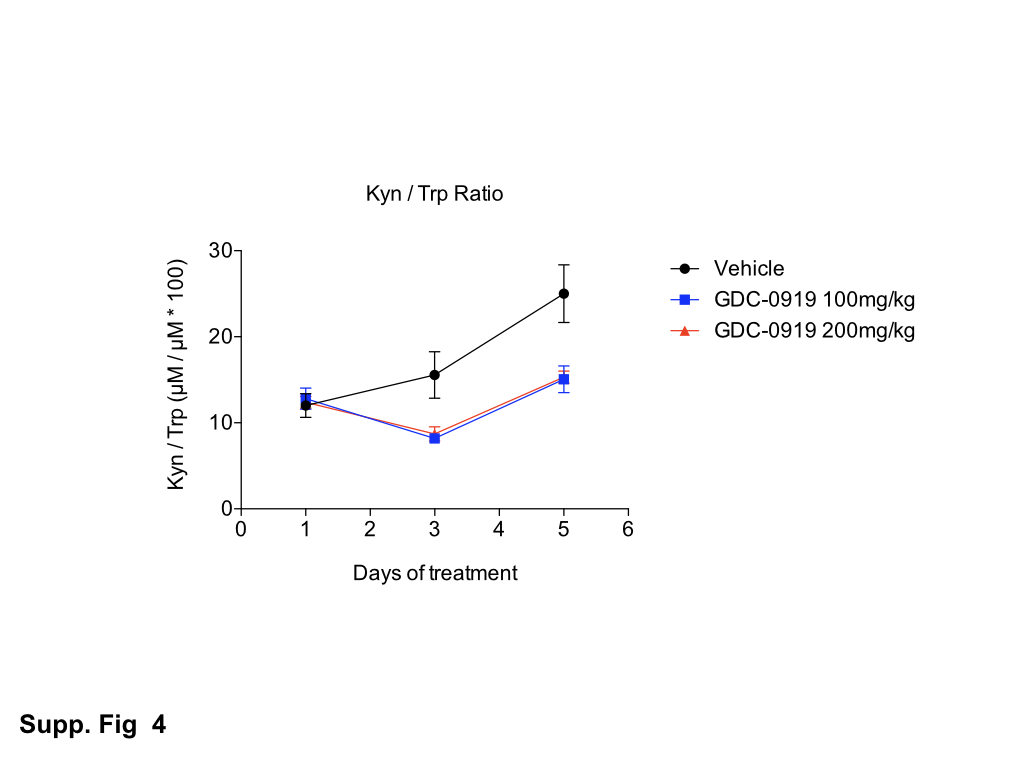

Supplement: Supplementary file 4 [file Image_4.tiff]

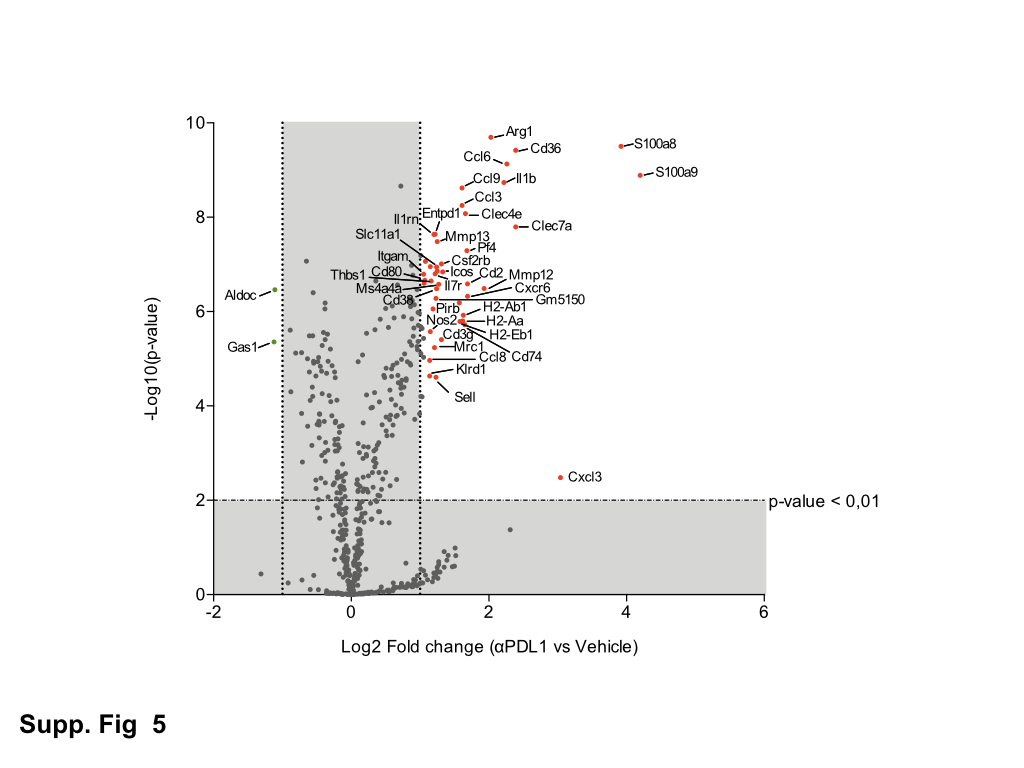

Supplement: Supplementary file 5 [file Image_5.tiff]
