# Supplementary material for: IDO Targeting in Sarcoma: Biological and Clinical Implications
Source: Front Immunol. 2020 Mar 5;11:274. doi: 10.3389/fimmu.2020.00274 (PMC7066301; doi:10.3389/fimmu.2020.00274)
Supplement: Supplementary file 6 [file Data_Sheet_1.PDF]

IDO targeting in sarcoma: biological and clinical implications

Imane NAFIA[1], Maud TOULMONDE[2], Doriane BORTOLOTTO[1], Assia CHAIBI[1], Dominique BODET[1], Christophe REY[1], Valerie Velasco[2], Claire LARMONIER[2], Loïc CERF[1], Julien ADAM[3], François LE LOARER[2], Ariel SAVINA[4], Alban BESSEDE[1]*†, Antoine ITALIANO[2,5]*†

[1]Explicyte Immuno-Oncology, Bordeaux, France

[2]Department of Medical Oncology, Institut Bergonié, Bordeaux, France

[3]Department of Pathology, Gustave Roussy, Villejuif, France

[4]Institut Roche, Paris, France

[5]Inserm U1218, Bordeaux, France

***Correspondence**

a.italiano@bordeaux.unicancer.fr

a.bessede@explicyte.com

† These authors share senior authorship

Keywords: Sarcomas, Immunotherapy, Indoleamine, Kynurenine, PDL1

SUPPLEMENTARY MATERIALS

Supplementary Figure 1 - Kaplan-Meier curves of progression free survival (A) and overall survival (B) according to CD8+ immune cells infiltration (Median value as cutoff level) in STS patients.

Supplementary Figure 2 – Gene expression correlation between Ido1, Il6, and Ifng. Gene expression correlation of Ido1 *vs* Il6 & Ido1 *vs* Ifng in tumor samples collected from a MCA205 tumor-bearing mouse model treated or not with anti-PDL1. R^2 is mentioned in each case as an objective indicator of correlation.

Supplementary Figure 3 – Gene expression assessment in Tumor Draining Lymph Nodes (TDLNs) samples collected from a MCA205 tumor-bearing model treated or not with anti-PDL1. TDLNs were retrieved 13 days post tumor inoculation, and subjected to RT-qPCR for expression analysis of genes encoding for the different enzymes of the KP and key inflammatory cytokines. On the right bottom, display of the p-values vs. the log transformed ratio of tumor mRNA level between PDL1 and Vehicle. Means ± SEM are represented.

Supplementary Figure 4 –Assessment of plasmatic Tryptophan catabolism upon pharmacological inhibition of IDO1 in a LPS model. Naïve C57BL/6 mice were treated orally twice daily with GDC-0919 at 100 or 200mg/kg for 5 consecutive days and, one day after treatment initiation, LPS at 10mg/kg (*E.Coli*, O55:B5 serotype) was intraperitonealy administered. Before LPS injection and 2 and 4 days after, animals were bled, plasma collected and Kynurenine to Tryptophan ratio measured by mean of ELISA. Means ± SEM are represented.

Supplementary Figure 5 – Modulation of gene expression upon PDL1 blockade within tumors. Volcano plot of NanoString gene expression analysis in tumor samples comparing Vehicle vs anti-PDL1 (right panel). Dashed line depicts a 0,01 significativity.
